# Aliquis, in Answer to T.Y.

**Published:** 1807-08

**Authors:** 


### To Dr. BATTY.

Sir,

In perusing the lively correspondence of your animated writer, T. Y. in the last Number of your useful miscellany, I was, I confess, disappointed to find that his ingenious communication tended rather to afford a display of laconic wit, than any useful or important information. There is no subject, I conceive, which demands so much minute attention, and which we ought with equal assiduity to investigate, as the science of medicine; and I am fully convinced, that there is no pursuit so rational as an indefatigable attention to medical researches; but I fear that our ingenious correspondent has mistaken a speculative curiosity for a laudable desire of information from novel discoveries.

The "Ars medicinæ, is a subject which has engaged the attention of men of the first literary abilities, and those in general general to whom medicine is indebted for some of its most useful discoveries, have always treated it with that serious attention, which its acknowledged importance demands.— On whatever subject cheerfulness can be judiciously introduced, it will, (I allow) afford a lively air, and tend to unite agreeable amusement, with useful and necessary information. But whenever this vein of pleasantry is indulged beyond its proper limits, and converted into levity, it is, (I think) degrading a subject, upon which both private happiness and general welfare are dependant. I shall only offer a single observation with respect to the different modes of treatment adopted, and leave Dr. Kinglake and Dr. Kentish where T. Y. has left them, till some future opportunity occurs, to decide the difference between these zealous advocates for their respective Theories.

Improvement in medicine is a "*desideratum*" which would at all times be acceptable; but whenever any new *hypothesis* is introduced, I would recommend our correspondent in particular, and medical practitioners in general, not to desert the beaten path which experience has manifested to be beneficial, till they are convinced that the principle upon which they act, is as consistent as the treatment they adopt is successful. Towards the close of his communication, some phrases were made use of, which I thought beneath the importance of the subject. As for gonorrhœas being cured without any internal medicine, I say nothing, nor do I wish to dispute his word, having mentioned that personal experience had confirmed his assertion. But when he informs us, that "it is an even bet that port wine or scraped potatoes will heal a bruised forehead as readily as a solution of sal. ammoniac," he descends much below the dignity of the subject; and whilst he displays the fertile ingenuity of his own imagination, he strips medicine of all the honourable plumes of science, and makes it appear in no higher light than an ordinary mechanical subject.

If T. Y. is induced by these observations to recollect, (when he next appears in the Med. and Phys. Journal) that he is addressing men of science and erudition; to whom useful discoveries, or serious information, would be more acceptable than lively displays of eccentricity or animated turns of wit, the design of the writer will be fully accomplished.

ALIQUIS.

*July* 11, 1807.

